# The Apolipoprotein-AI Mimetic Peptide L4F at a Modest Dose Does Not Attenuate Weight Gain, Inflammation, or Atherosclerosis in LDLR-Null Mice

**DOI:** 10.1371/journal.pone.0109252

**Published:** 2014-10-06

**Authors:** Michelle M. Averill, Eung Ju Kim, Leela Goodspeed, Shari Wang, Savitha Subramanian, Laura J. Den Hartigh, Chongren Tang, Yilei Ding, Catherine A. Reardon, Godfrey S. Getz, Alan Chait

**Affiliations:** 1 Department of Environmental and Occupational Health, University of Washington, Seattle, Washington, United States of America; 2 Department of Medicine, University of Washington, Seattle, Washington, United States of America; 3 Department of Pathology, University of Chicago, Chicago, Illinois, United States of America; University of Amsterdam Academic Medical Center, Netherlands

## Abstract

**Objective:**

High density lipoprotein (HDL) cholesterol levels are inversely related to cardiovascular disease risk and associated with a reduced risk of type 2 diabetes. Apolipoprotein A-I (apoA-I; major HDL protein) mimetics have been reported to reduce atherosclerosis and decrease adiposity. This study investigated the effect of L4F mimetic peptide and apoA-I overexpression on weight gain, insulin resistance, and atherosclerosis in an LDL receptor deficient (*Ldlr^-/-^*) model fed a high fat high sucrose with cholesterol (HFHSC) diet.

**Methods:**

Studies in differentiated 3T3-L1 adipocytes tested whether L4F could inhibit palmitate-induced adipocyte inflammation. *In vivo* studies used male *Ldlr^-/-^* mice fed a HFHSC diet for 12 weeks and were injected daily with L4F (100 µg/mouse) subcutaneously during the last 8 weeks. Wild-type and apoA-I overexpressing *Ldlr^-/-^* mice were fed HFHSC diet for 16 weeks.

**Results:**

Neither L4F administration nor apoA-I overexpression affected weight gain, total plasma cholesterol or triglycerides in our studies. While pre-treatment of 3T3-L1 adipocytes with either L4F or HDL abolished palmitate-induced cytokine expression *in vitro*, L4F treatment did not affect circulating or adipose tissue inflammatory markers *in vivo*. Neither L4F administration nor apoA-I overexpression affected glucose tolerance. ApoA-I overexpression significantly reduced atherosclerotic lesion size, yet L4F treatment did not affect atherosclerosis.

**Conclusion:**

Our results suggest that neither L4F (100 µg/day/mouse) nor apoA-I overexpression affects adiposity or insulin resistance in this model. We also were unable to confirm a reduction in atherosclerosis with L4F in our particular model. Further studies on the effect of apoA-I mimetics on atherosclerosis and insulin resistance in a variety of dietary contexts are warranted.

## Introduction

The strong inverse relationship between high density lipoprotein (HDL) cholesterol levels and coronary heart disease [1] has been known for over three decades. HDL's role in reverse cholesterol transport has been hypothesized to inhibit atherogenesis [2], and has been thought to contribute to HDL's role as a negative cardiovascular risk factor. Recent research also has highlighted other functional properties of HDL, including its anti-inflammatory and anti-oxidant activities, as additional mechanisms to reduce atherosclerosis [3]. Low HDL cholesterol levels are also associated with development of type 2 diabetes mellitus (T2DM) [4]. Inflammation has been suggested to be responsible for the reduction in HDL levels seen in the setting of insulin resistance and obesity [5]. However, recent data have also highlighted anti-inflammatory properties of HDL [6], suggesting HDL may improve insulin sensitivity [7].

Apolipoprotein A-I (apoA-I) is the main protein in HDL and contributes to the cholesterol efflux, anti-oxidant, and anti-inflammatory potential of HDL [2]. ApoA-I mimetic peptides are 18 amino acid peptides modeled after the amphipathic α-helical structure of apoA-I [8]. The 4F peptide, the most widely studied mimetic, has been shown to promote cholesterol efflux, have anti-inflammatory properties, and to reduce atherosclerosis and adiposity in some murine models [8]–[10]. Early phase trials of 4F use in humans have been reported [11]. The majority of murine studies with the 4F mimetic peptide have been conducted in apolipoprotein E deficient (*apoE^-/-^*) mice, a common model of hyperlipidemia associated with development of premature atherosclerosis [9]. The *apoE^-/-^* model does not lend itself to the study of obesity or insulin resistance, whereas the *Ldlr^-/-^* mouse, another common model of atherosclerosis, allows for the combined study of diet induced obesity, insulin resistance, and atherosclerosis [12]. Thus further investigation of the effect of the 4F mimetic peptide in the *Ldlr^-/-^* model is warranted.

The purpose of the current study was to determine the effect of 4F mimetic peptide on atherosclerosis and obesity in *Ldlr^-/-^* mice fed an obesogenic diet. We also determined the effect of either apoA-I overexpression or the 4F mimetic peptide on insulin resistance and inflammation. We used a previously validated model of diet-induced obesity (DIO) to induce atherosclerosis and insulin resistance, i.e., male *Ldlr^-/-^* mice fed a high fat high sucrose diet with added cholesterol (HFHSC) [13]. The L4F version of the peptide, synthesized using L amino acids, was used based on our previous studies [10].

## Methods

### L4F Mimetic Peptides

N-terminally acetylated and C-terminally amidated L4F peptide was purchased from BioSynthesis. The “4F” denotes the following peptide: Ac-DWFKAFYDKVAEKFKEAF-NH2. Lyophilized peptide was stored at −20°C and solubilized in sterile PBS immediately prior to use. The L4F peptide does not form precipitates upon extended storage at 4°C in PBS [14].

### *In Vitro* Adipocyte Studies

The effect of HDL and L4F peptide on adipocyte inflammation was determined using 3T3-L1 adipocytes exposed to palmitate. HDL (d = 1.063 to 1.210 g/mL) was isolated from plasma of healthy human volunteers by ultracentrifugation. 3T3-L1 murine pre-adipocytes (American Type Tissue Culture Collection) were differentiated into adipocytes according to standard procedures [15]. Fully differentiated 3T3-L1 adipocytes, cultured in DMEM media containing 5 mmol/L glucose and 10% fetal bovine serum, were pre-treated with 50 µg/ml HDL or 25 µg/ml L4F for 6 h. After the pre-treatment, cells were washed three times with PBS. Adipocytes were incubated with 250 µmol/L palmitate (16:0; Sigma) for 24 h (DMEM media containing 5 mmol/L glucose and 10% fetal bovine serum), as described previously [6]. At the end of the study RNA was isolated from the 3T3 cells using a lipid specific RNA isolation kit (Qiagen) and gene expression was determined using RT-PCR.

### Real-time quantitative reverse-transcription polymerase chain reaction (RT-PCR)

Specific gene expression was determined using specific primers and TaqMan probes obtained from Applied Biosystems Assay-on-Demand and the TaqMan Master kit (Applied Biosystems). GAPDH was used as the control housekeeping gene. Samples were run using the ABI prism 7900HT system. Each sample was analyzed in triplicate and relative amounts of target genes calculated using the ΔΔCT formula.

### Animals and Diet

Male *Ldlr^-/^* ^-^ mice originally purchased from Jackson Labs (Sacramento, CA, USA) on the C57BL/6 background, were 10-weeks-old when started on a HFHSC diet (35.5% calories as fat and 36.6% as carbohydrate, 0.15% added cholesterol, BioServ No. F4997). Mice used for the mimetic study were on diet for 12 weeks, an early time point chosen to model our previous data showing the L4F peptide works on early rather than on advanced lesions [10]. After 4 weeks of diet, either saline or L4F peptide (100 µg/mouse) was injected subcutaneously daily for the remaining 8 weeks of the study, the injection duration used in our previous study [10].

*ApoA-I^tg^* (mice overexpressing human apoA-I) mice were purchased from the Jackson laboratory. *Ldlr^−/−^apoA-I^tg^* and control *Ldlr^−/−^apoA-I^wt^* mice were generated from these homozygous mice. Confirmation of apoA-I overexpression was done using quantitative RT-PCR to confirm homozygous overexpression and an ELISA for human apoA-I confirmed appropriate genotype for all mice used in breeding and in the study. From this breeding colony adult (10-week-old) male *Ldlr^-/-^* mice overexpressing human apoA-I (*apoA-I^tg^*) and control *Ldlr^-/-^* mice (*apoA-I^wt^*) were fed the HFHSC diet for 16 weeks. A longer time on diet was chosen to analyze more advanced stages of atherosclerosis and more dramatic differences in insulin sensitivity, corresponding with several of our recent studies [16]. The *apoA-I^tg^* mice are the same as reported previously [6]. Data in this current manuscript is additional to that previously reported. However a limited amount of data from that report is reproduced here for direct comparison of the response with that seen after peptide treatment.

At sacrifice, epididymal adipose tissues were snap-frozen at −70°C for isolation of total RNA using lipid specific isolation kit (Qiagen). Aortic tissue were excised at the time of sacrifice and prepared for atherosclerosis analysis (described below).

### Ethics Statement

All experimental procedures were undertaken with approval from the Institutional Animal Care and Use Committee of the University of Washington (Protocol Number: 3104-01). The study was carried out in accordance with the National Institutes of Health recommendations based on the published *Guide for the Care and Use of Laboratory Animals* [17]. All efforts were made to minimize animal suffering.

### Analytic Procedures for Mimetic and apoA-I overexpression study

At both 4 weeks into the diet and the end of each study blood was collected after a 4–6 hour fast. Analysis of plasma insulin was done using a commercially available kit (Linco Research Inc). Plasma triglycerides and cholesterol were assayed using colorimetric assay kits, and levels of lipoproteins were analyzed by fast-phase liquid chromatography (FPLC) as described previously [13]. Plasma SAA levels (isoforms 1 and 2) were measured by enzyme-linked immunosorbent assay (ELISA) [13]. Glucose tolerance testing (GTT) was performed as previously described [13] at week 11 in mimetic study and week 14 in apoA-I overexpression study. Plasma was collected at the 30 minute time point of the GTT to analyze glucose stimulated insulin levels. Body composition was determined in the mimetic study using quantitative magnetic resonance (qMRI) (EchoMRI 3-in-1 machine whole body composition analyzer; Echo MRI, LLC, Houston, TX) after 10 weeks on diet [18], [19].

### Atherosclerosis Quantification

The en face technique was used to quantify the extent of atherosclerosis as described previously [20]. Additional atherosclerosis quantification was done in the mimetic study by analyzing atherosclerosis in the brachiocephalic artery [21]. The brachiocephalic artery was dissected, fixed, embedded, and serial sectioned. Every fourth section was stained with a Movat pentachrome stain. Atherosclerotic lesion area in the brachiocephalic artery was determined in a masked fashion by calculating maximal and average lesion area.

### Statistical analysis

Data shown are expressed as means± standard deviation unless noted otherwise. With the exception of atherosclerotic lesions data, mean values were compared using ANOVA and post-hoc testing with Bonferroni correction for multiple comparisons for parametric data using GraphPad Prism program (Version 3.03, GraphPad Software Inc). As the atherosclerotic lesion data was not normally distributed (D'Agostino and Pearson omnibus normality test) this analysis employed the Mann-Whitney non-parametric test. Significance was denoted at p-value less than 0.05.

## Results

### L4F inhibits palmitate induced chemokine expression in 3T3-L1 adipocytes

**Figure 1:**
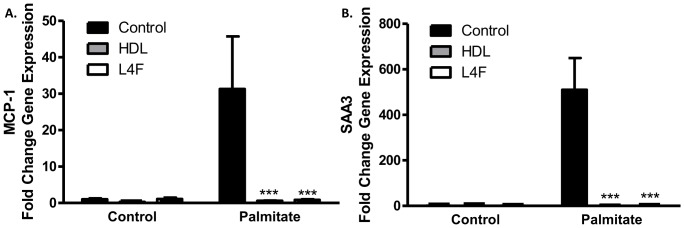
L4F mimetic peptide blocks palmitate-induced chemokine factor expression in 3T3-L1 adipocytes. MCP-1 (A) and SAA3 (B) gene expression in 3T3-L1 cells exposed to control (fatty acid free albumin) or palmitate (250 µmol/l). Prior to fatty acid exposure cells were pre-incubated with control, HDL (50 µg/ml), and L4F (25 µg/ml) containing media. Fold gene expression was determined using the ΔΔCT fold change method normalized to GAPDH. Each graph is representative of three independent experiments. ***P<0.001 vs control in palmitate treated cells using 2-way ANOVA and Bonferroni adjustment for multiple comparisons.

We previously have shown that palmitate induces chemotactic factor expression in 3T3-L1 adipocytes [15] and this can be inhibited by pre-treatment with HDL [6]. Thus, we investigated if L4F peptide can similarly inhibit chemokine expression in 3T3-L1 cells. After palmitate exposure 3T3-L1 cells pre-treated with L4F peptide expressed significantly less MCP-1 and SAA3 (figure 1), two chemokines present in inflamed obese adipose tissue [13]. This effect was comparable to that seen with HDL pre-treatment.

### Neither L4F (100 µg/day/mouse) nor apoA-I overexpression attenuate weight gain in *Ldlr^-/-^* mice fed HFHSC diet

**Figure 2:**
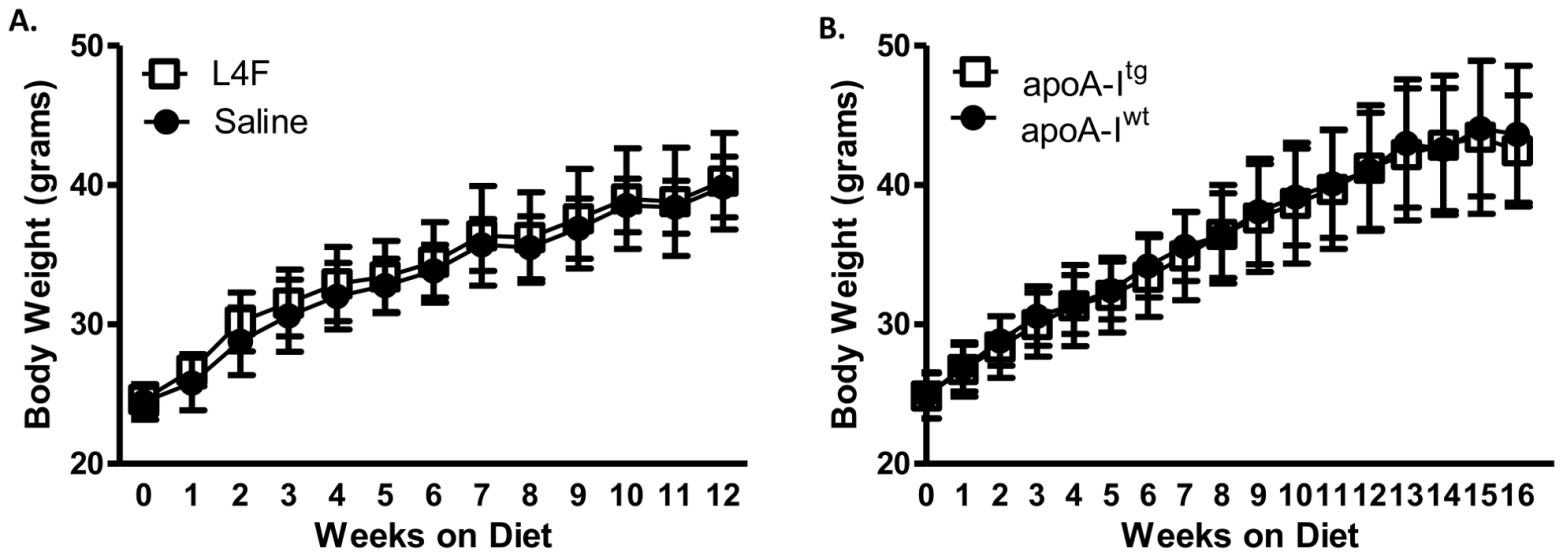
100 µg/day/mouse L4F mimetic peptide and apoA-I overexpression do not affect weight gain. Body weights for the peptide study (A; n = 5) and the apoA-I overexpression study (B; n = 15–17). No significant differences were identified.

The diet employed in this study is one intended to induce a large gain in weight. Treatment of *Ldlr^-/-^* mice with L4F peptide given at 100 µg/day/mouse had no impact on the extent of weight gain (figure 2A), compared with the saline injected animals. Further, there was no impact of L4F treatment on fat mass measured by qMRI (36.4%±3.3% saline versus 35.6%±5.1% L4F). Similarly, gain in body weight was no different for human apoA-I overexpressing mice on the *Ldlr^-/-^* background than in mice expressing murine apoA-I at wild type levels (figure 2B). Also the weight gain was very similar for the *Ldlr^-/-^* animals in the mimetic study and the apoA-I study over the first 12 weeks of diet.

### Neither L4F (100 µg/day/mouse) nor apoA-I overexpression affected plasma total cholesterol or triglycerides

**Figure 3:**
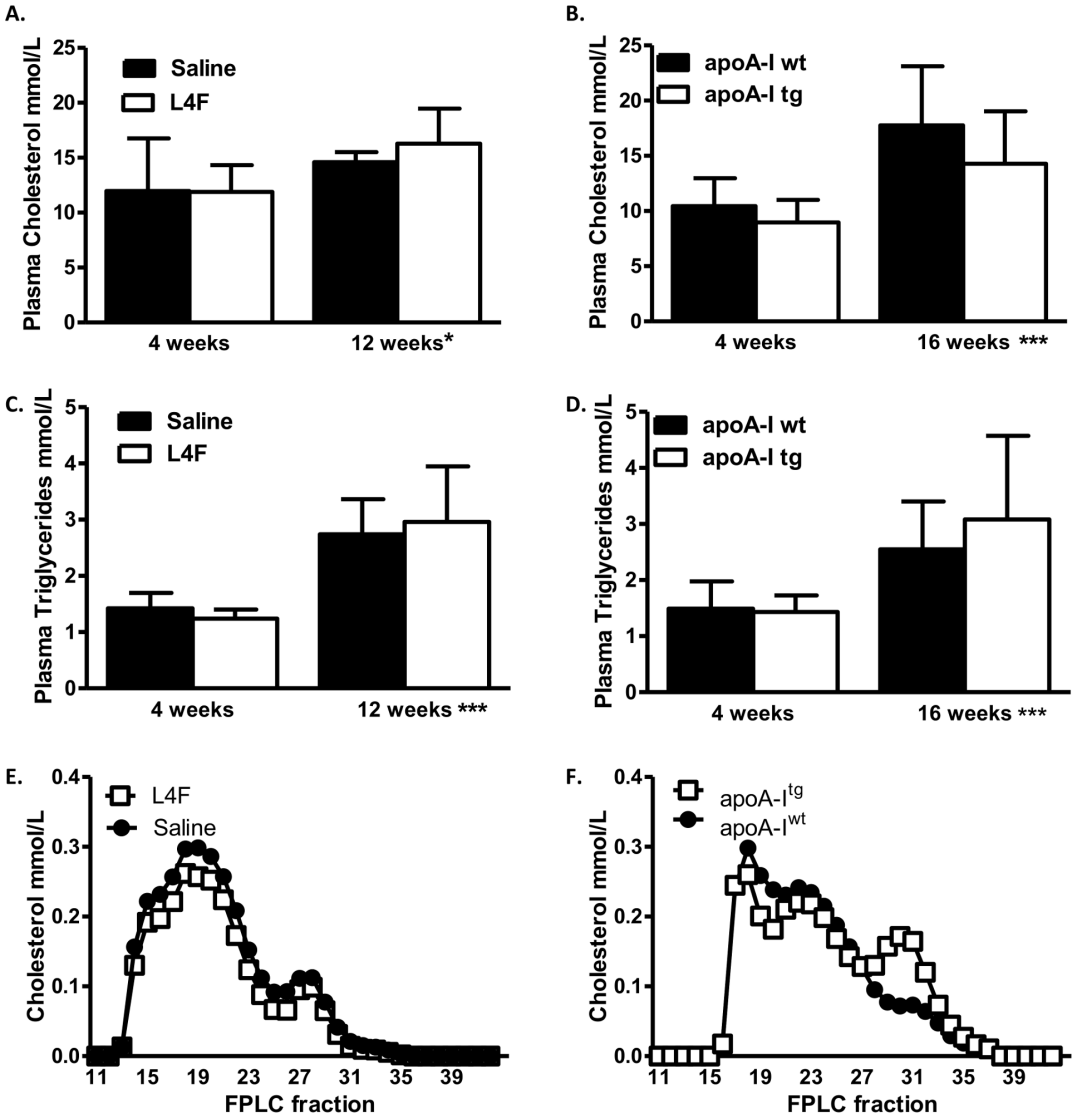
100 µg/day/mouse L4F mimetic peptide and apoA-I overexpression do not affect plasma total cholesterol or triglycerides. Total plasma cholesterol and triglycerides are shown for the peptide (A,C; n = 5) and the apoA-I overexpression study (B,D, n = 9–13) after 4 weeks on the diet and at the end of the study protocol. FPLC cholesterol analysis on pooled plasma samples from the peptide study (E) and the apoA-I overexpression study (F). Differences in cholesterol eluting times, as noted by the different starting peaks for cholesterol, are due to a newer FPLC column used with the apoA-I overexpression study. No significant differences were identified. *P<0.05 4 week versus 12 weeks on diet peptide study, ***P<0.0001 4 week vs 16 weeks on diet apoA-I study by paired two-way ANOVA.

The *Ldlr^-/-^* HFHSC fed mouse develops hyperlipidemia with increased VLDL and LDL cholesterol, which promotes atherosclerosis [22]. However, in *Ldlr^-/-^* mice fed the HFHSC diet, neither L4F nor apoA-I overexpression affected plasma total cholesterol or triglyceride levels (figure 3 A through D). There were also no differences noted in plasma cholesterol or triglycerides after 4 weeks on diet (figure 3 A through D), a time point chosen as the baseline before the initiation of peptide injections. In both the peptide study and the apoA-I overexpression study plasma cholesterol and triglyceride did significantly increase over the remaining time on diet (figure 3 A through D). There was no indication that L4F alters the lipoprotein profile (figure 3E). FPLC profiles do suggest apoA-I overexpression increases HDL cholesterol content (figure 3F), yet we were unable to assess the statistical significance of this difference since the FPLC profiles represent pooled samples.

### L4F (100 µg/day/mouse) did not attenuate systemic or adipose tissue inflammation

**Figure 4:**
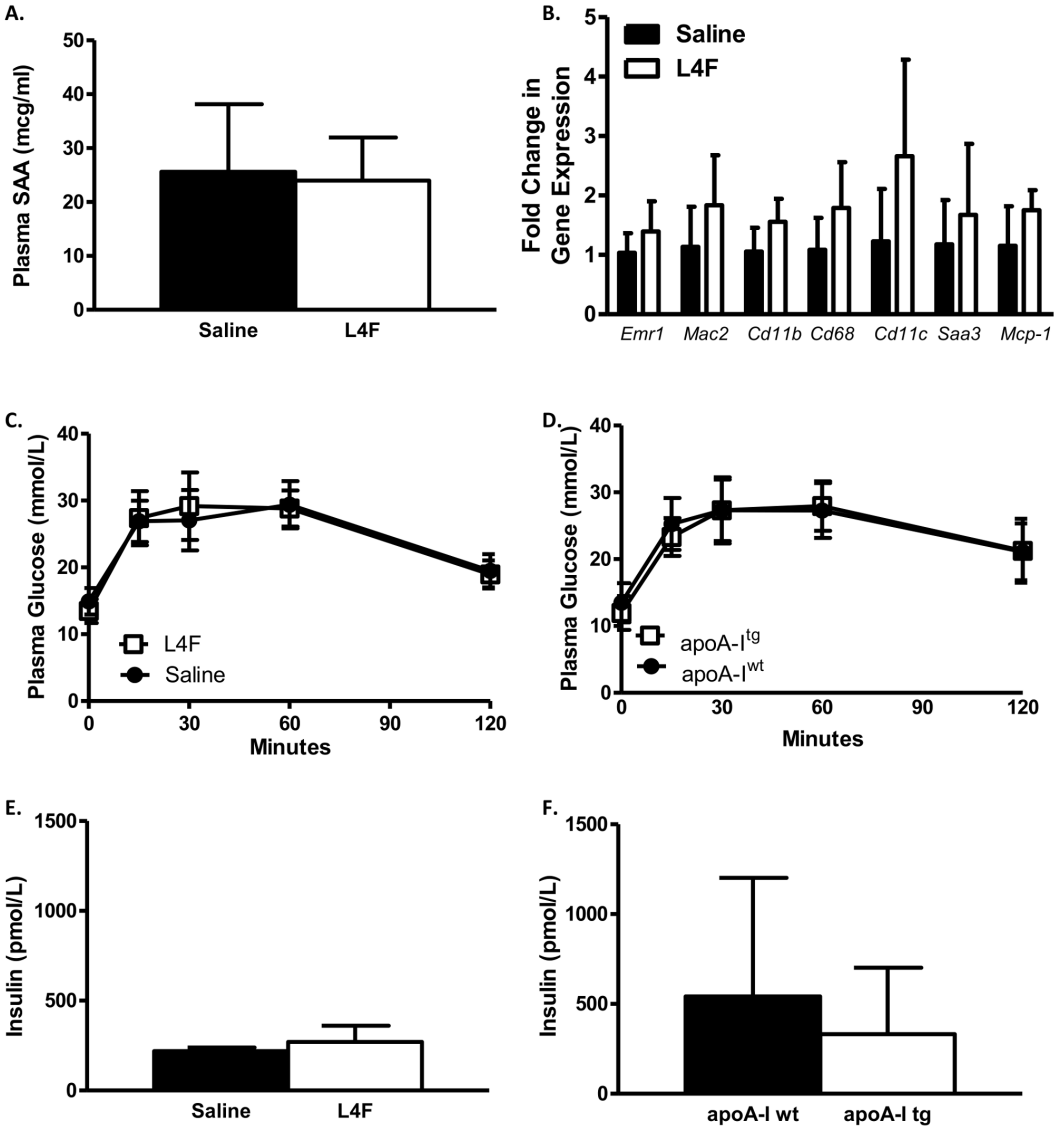
100 µg/day/mouse L4F did not attenuate inflammation, and neither L4F nor apoA-I overexpression improved glucose tolerance. SAA was measured in the plasma (A, n = 5) and inflammatory gene expression was analyzed in epididymal adipose tissue extracts (B, n = 4–5). GTTs are shown for the peptide study (C: n = 5) and the apoA-I overexpression study (D: n = 10–12). Plasma was collected at the 30 minute time point of the GTT and insulin was measured in both studies (E, F). No significant differences were noted.

One mechanism potentially linking obesity to insulin resistance is through adipose tissue inflammation. We have shown in previous studies that the HFHSC diet in *Ldlr^-/-^* mice results in increased macrophage content in adipose tissue and increased expression of inflammatory cytokines [13]. We have also shown that overexpression of apoA-I in C57BL/6 mice or *Ldlr^-/-^* mice inhibit adipose tissue macrophage content and inflammatory gene expression [6]. Given that L4F elicits an anti-inflammatory response in 3T3-L1 cells we examined the effect of L4F on SAA, a marker of systemic inflammation and on adipose tissue inflammatory gene expression. In contrast to our previous results in the apoA-I overexpression model [6], we did not see any effect of L4F (100 µg/day/mouse) on circulating SAA, or adipose tissue gene expression (figure 4A and B).

### Neither L4F (100 µg/day/mouse) nor apoA-I overexpression improve glucose tolerance in *Ldlr^-/-^* mice fed HFHSC diet

Our original hypothesis was that the L4F mimetic would decrease adipose tissue inflammation and thus improve glucose homeostasis. In order to test this, glucose tolerance tests and glucose-stimulated insulin levels were examined. There was no significant effect of L4F peptide given at 100 µg/day/mouse on glucose tolerance or glucose-stimulated insulin secretion (figure 4C and E). As with the response to L4F, apoA-I overexpression did not improve glucose tolerance (figure 4D). In addition, there was no effect of apoA-I overexpression on glucose-stimulated plasma insulin levels (figure 4F).

### L4F (100 µg/day/mouse) did not attenuate atherosclerosis

**Figure 5:**
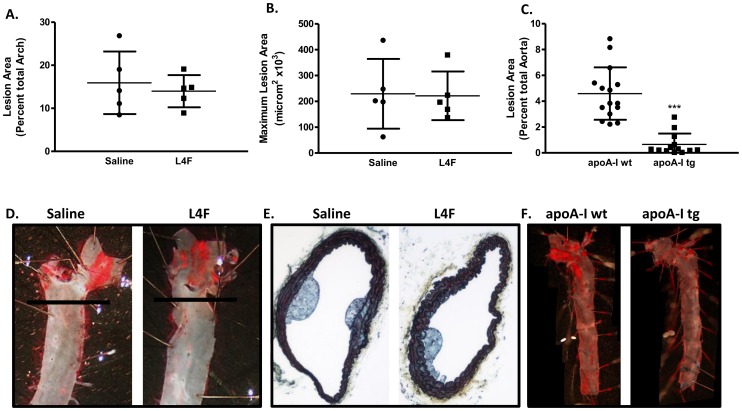
100 µg/day/mouse L4F did not while apoA-I overexpression did reduce atherosclerosis, in *Ldlr^-/-^* mice fed HFHSC diet. Atherosclerotic lesion area was determined using the *en face* method. Given the small lesion size the percent lesion area was calculated for the arch section in the peptide study (A, n = 5) and calculated as a percent of the total aorta in the apoA-I overexpression study (C, n = 13-15). Analysis of the maximum lesion area in the brachiocephalic artery lesions is shown for the peptide study (B, n = 5). Representative lesions are shown for the peptide study for both the en face (D) and the brachiocephalic artery (E). Note the black line in the en face image (D) indicated the bottom of the arch indicating the boundary of the analysis. Representative en face images for the apoA-I study are also shown (F). ***P<0.0001 vs apoA-I wt by Mann-Whitney.

Our final objective was to determine if L4F peptide could inhibit atherosclerosis similarly in the *Ldlr^-/-^* model as has been described for the *ApoE^-/-^* model. We examined the extent of atherosclerosis using the *en face* method as previously described [20]. Given the small size of the lesions our final analysis was based only on the arch and not the entire aorta for the mimetic study. Our results suggest there was no effect on lesion size with the L4F mimetic provided at 100 µg/day/mouse in *Ldlr^-/-^* mice (figure 5A, representative images in figure 5D). We also assessed lesion area in the brachiocephalic artery and found no difference in maximum lesion area (figure 5B) or average lesion area (data not shown). Morphological assessment of these lesions revealed the lesions were characterized by foam cell accumulation with no evidence of fibrosis or necrotic core formation, characteristic of early fatty streaks. No differences in lesion morphology were identified and representative images are shown in figure 5E. To validate our model and methods, the effect of apoA-I overexpression on lesion area was examined by the *en face* method. As expected and supporting previous research [23], a significant reduction in atherosclerosis was observed (figure 5C; representative images in figure 5F). It is important to note that the absolute extent of lesions differed between the mimetic and apoA-I overexpression studies due to the fact that the mimetic study focused on the arch while the apoA-I study included the entire aorta.

## Discussion

In this manuscript we report several new findings. Our results suggest that while L4F can markedly reduce adipocyte inflammation *in vitro* similar to HDL, this does not carry through to improvements in inflammation, glucose tolerance, or atherosclerosis in *Ldlr^-/—^*male mice fed the HFHSC diet using an LF4 dose of 100 µg/day/mouse. Further, despite our previous studies showing that apoA-I overexpression can reduce adipose tissue inflammation, our current data suggests this does not confer improvements in glucose tolerance.

Our *in vitro* studies demonstrated a novel ability of L4F to mimic apoA-I function in adipocytes. ApoA-I [6], HDL and L4F (figure 1) exposure markedly reduced the expression of inflammatory markers in 3T3L1 cultured adipocytes. The dramatic efficacy of L4F in these *in vitro* experiments suggests that the peptide is functioning in culture in a similar fashion to apoA-I in HDL. As the L4F peptide was developed to mimic apoA-I function, and this appears to be borne out by our *in vitro* studies, we have compared apoA-I overexpression with L4F treatment in several pathophysiological endpoints in *Ldlr^-/—^*male mice fed the HFHSC diet. The effect of apoA-I overexpression on insulin resistance in the *Ldlr^-/-^* DIO model has not been reported on previously, and thus an important dataset for comparison to L4F treatment. Provided chronic inflammation which is associated with obesity related insulin resistance [24], our *in vitro* findings suggest that L4F and apoA-I overexpression could potentially improve glucose tolerance in an *in* vivo model of DIO. In our previous study apoA-I overexpression relieved some of the adipose tissue inflammation [6]. However, in the present study L4F treatment of 100 µg/day/mouse had no impact on adipose tissue or systemic inflammatory markers (figure 4). Further, neither the overexpression of apoA-I nor treatment with L4F had an impact on glucose tolerance nor glucose stimulated insulin levels (figure 4). Note that neither apoA-I overexpression nor L4F treatment had an impact on weight gain or adiposity in this model (figure 2), confirmed in the peptide study by body composition analysis. ApoA-I and L4F mimetics are often cited for their anti-atherosclerotic properties. This effect of apoA-I overexpression in *Ldlr^-/-^* mice fed a high fat diet has been documented [23] and confirmed in the present study (figure 5). Yet, L4F treatment had no such effect at a dosage of 100 µg/day/mouse.

It is striking that despite the dramatic effect of L4F on 3T3L1 cells, the peptide is without influence on the measured parameters studied *in vivo* at 100 µg/day/mouse. One limitation of this study is that the absence of any *in vivo* effect of peptide treatment raises questions about the efficacy of the dose and route of administration of the peptide. We have followed protocols and dosages that have been shown in *apoE^-/^* ^-^ and *ob/ob* mice to be effective [10], [25]. The dose we employed (4–5 mg/kg/day), is equal to the effective dose of 4.5 mg/kg/day shown by Navab and colleagues to reduce plasma SAA levels [26]. Importantly, they have found the dose was determining rather than the concentration in the plasma [26]. This group has further suggested that the primary function of the peptides is to attenuate the oxidized lipid and unsaturated lysophosphatidic acid concentrations in the intestine [27]. The current study utilized the L version of the peptide injected subcutaneously as it is not stable for oral delivery while other studies have used the D version of the peptide which is stable for oral delivery. However, data suggests that neither the route of administration nor the enantiomer version of the peptide determines effectiveness. One study directly compared L4F versus D4F delivered subcutaneously in rabbits and found similar reductions in atherosclerosis [28]. While studies suggest the site of action is primarily in the intestine, Navab and colleagues have shown D4F is similarly effective when delivered subcutaneously or orally and equivalent amounts appear in the feces [29]. L4F delivered orally with niclosamide (which protects the peptide from degradation) was equally effective in reducing SAA in *apoE^-/-^* mice as L4F delivered subcutaneously [26]. This suggests that the effect of 4F peptide on inflammation and atherosclerosis is independent of route of administration and the enantiomer version of the peptide, and thus does not explain the lack of effect in the current study. Given the above discussion, it is possible that either L4F does not influence obesity, inflammation, or atherosclerosis in this model or that a higher dose of peptide is required to affect these outcomes in this model. In addition, we have no information on the role of oxidized lipids in the intestine in our model. Further work is needed to address the effectiveness of 4F peptide, optimal dosage, route of administration, and potential mode of action in *Ldlr^-/—^*male mice fed the HFHSC diet.

Another recognized limitation of our study is the small sample size in the peptide group (n = 5). To determine the extent to which this limitation impacts the ability to draw conclusions from the data power analysis and sample size calculations were performed (Graphpad StatMate 2.00). The sample size calculation was based on the observed standard deviation of 5.54% for the percent of total arch that contains lesion and an observed difference in the means of 2%. Considering these statistics and a desired power>80%, a sample size of greater than 100 per group would be needed to identity significant differences. Based on these sample size calculations it is not expected that doubling or tripling the sample size of the current study would result in significant differences.

Most studies with mimetic peptides have been performed with *apoE^-/^* ^-^ mice, which has been responsive in several respects to L4F treatment, especially on early atherosclerotic lesions [10]. Relatively few experiments have addressed the role of the peptide on inflammation and atherosclerosis in *Ldlr^-/-^* mice. Of the studies investigating the effect of 4F peptide in *Ldlr^-/-^* mice, the dosages used which resulted in a reduction of inflammation or atherosclerosis were 9–25 times greater than the dosages employed in the current study [30], [27], [31]. Thus it is possible that *Ldlr^-/-^* mice are less sensitive to peptide treatment than are *apoE^-/-^* mice and a higher dose may be required to observe reductions in inflammation and atherosclerosis. Possible contributors to reduced sensitivity to peptide in this model may be not only the absence of the LDLR but also the interaction with the highly obesogenic diet.

With respect to adiposity and glucose tolerance, one study used chow-fed ob/ob mice injected with L4F at the dose of 2 mg/kg/day and demonstrated attenuated obesity and adipose inflammation [25], [32]. In that study, L4F treatment also improved both glucose and insulin tolerance tests. While the ob/ob model consistently develops obesity related insulin resistance, the hyperlipidemia is characterized by elevations in HDL and this does not associate with atherosclerosis development [12]. We are the first to have reported effects on weight gain, inflammation, insulin resistance, and atherosclerosis in a model of DIO with lipid profiles similar to humans [12]. Our ability to compare the effects of L4F treatment directly to that of apoA-I overexpression is another strength of this research. We have shown using the HFHSC diet in C57BL/6 mice that were either wild type or transgenic with respect to apoA-I expression, apoA-I overexpression reduced adipose tissue inflammation [6]. Similar reductions of adipose tissue inflammation by apoA-I overexpression were also seen in the *Ldlr^-/-^* background [6], with no change in total plasma cholesterol or triglyceride levels. It is generally thought that adipose tissue inflammation is related to several systemic perturbations, such as glucose intolerance [24]. In the present study, despite the reduction of adipose tissue inflammation, apoA-I overexpression did not influence weight gain or glucose sensitivity in the DIO *Ldlr^-/-^* male model. This suggests that the gain of adipose tissue mass is not strictly correlated with the degree of inflammation in this tissue. These data argue that the relationship between adiposity, weight gain, adipose tissue inflammation, and glucose intolerance is more complex than has been thought and are not directly correlated. Peptide treatment of *Ldlr^-/-^* mice on the HFHSC diet also did not influence glucose tolerance [24] at a moderate dose of 100 µg/day/mouse.

Peptide treatment could have two major actions: influence on cholesterol homeostasis and reduced oxidized lipid concentrations [9]. In our model, there is no evidence of these actions of peptide being operative *in vivo*. Perhaps the drive of our dietary model toward atherogenesis and glucose intolerance is so strong as to be beyond the reach of the peptide at the level we employed, which was dosed based on studies in *apoE^-/-^* and *ob/ob* mice [10], [25]. It is not clear whether obesity in this model would indeed be responsive to higher doses of peptide. While a majority of the studies have been conducted in *apoE^-/-^* mice, this model is driven by hyperlipidemia and does not lend itself to investigation of several other aspects of the metabolic syndrome, namely DIO and insulin resistance. Further study in models that incorporate obesity and insulin resistance, including the *Ldlr^-/-^* mice fed a HFHSC diet, will improve our understanding of the potential to utilize mimetic peptides in all aspects of the metabolic syndrome.

There continues to be a strong relationship between increased apoA-I and HDL in animal models and reduced atherosclerosis. However, the true potential of mimetic peptides as a means to harness the positive properties of apoA-I remain uncertain. Further, there is some question as to the most appropriate model of insulin resistance and atherosclerosis and it is likely studies in multiple models are needed to identify the true potential of mimetics. Results presented in the current paper do not support a protective role for apoA-I mimetic L4F at a dose of 100 µg/day/mouse in weight gain, inflammation, insulin resistance, or atherosclerosis in a model of DIO (*Ldlr^-/-^* male mice fed HFHSC). Future research needs to verify the mechanism of protection and identify an appropriate dosage, route of delivery and timing to effect change on atherosclerosis progression in appropriate models of human disease. The ability for HDL to prevent insulin resistance remains an intriguing idea, and despite results in the current model it warrants further research.
